# A Novel Technique Debulking Vegetations in Tricuspid Endocarditis and Venacava Utilizing AngioVac Aspiration System

**DOI:** 10.7759/cureus.22283

**Published:** 2022-02-16

**Authors:** Nitish Mittal, Rohan Mittal, Mikal C Ramon, Zhaunn Sly, Mohammad M Ansari

**Affiliations:** 1 Internal Medicine/Cardiology, Texas Tech University Health Sciences Center, Lubbock, USA

**Keywords:** endocarditis, superior vena cava, tricuspid valve, vegetations, angiovac

## Abstract

The AngioVac system (AngioDynamics Inc., Latham, NY) is used for the removal of commonly encountered intravascular material, such as thrombus or vegetations in the right atrium, right ventricle, superior vena cava, and inferior vena cava. Patients with high surgical risk having tricuspid endocarditis and superior vena cava thrombus can be treated with the AngioVac system, hence mitigating the risks for this patient population. We present a case series with the utilization of the AngioVac device to reduce the vegetation size and decrease the risk of emboli with effective antibiotic penetration. Transesophageal echocardiography shows a reduction in the size of the vegetations in all three cases with no postoperative complications. This case series demonstrates a novel technique debulking vegetations in tricuspid endocarditis and vena cava.

## Introduction

The AngioVac system (AngioDynamics Inc., Latham, NY) consists of two entities: the AngioVac Cannula and AngioVac Circuit. This new directed suction catheter device is employed to perform percutaneous thrombo-embolectomy with decreased mortality rate [1]. It is composed of a cannula and extracorporeal circuit with a filter for pump-assisted removal of intravascular debris [2]. The system also features a self-expanding nitinol funnel-shaped tip that enhances drainage flow and prevents clogging of the cannula [3]. Direct imaging with echocardiography is utilized for successful catheter deployment to target thromboembolism while simultaneously avoiding iatrogenic injury [4].

Right-sided native valve infective endocarditis (IE) involves the tricuspid and pulmonic valves; right-side IE accounts for approximately 10% of all the IE cases [5]. Some common risk factors include injection drug use, infected pacemaker leads of a cardiac implantable electronic device, presence of intravascular devices, such as central line or intra-aortic balloon pump, and underlying right-sided cardiac anomaly [6]. *Staphylococcus aureus* is the most common microbe accounting for 70% of the infections, followed by Streptococci, Enterococcus, and *Pseudomonas aeruginosa* [7]. In all IE cases, surgical consultation is warranted, especially with large vegetations and recurrent infections. However, patients are considered at high risk for surgery in some instances due to severe valve disease. That is what transforms this case series into an informative application of the AngioVac device in the setting of contraindication for the surgical approach.

## Case presentation

Case one

**Figure 1:**
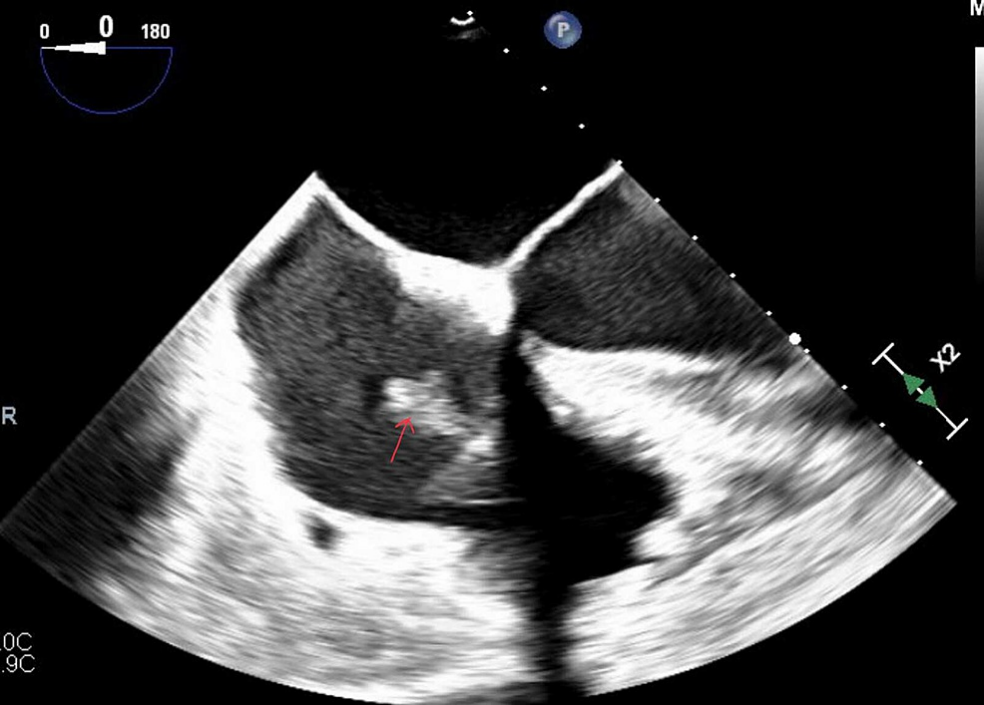
TEE showing the vegetations on tricuspid valve: 3 × 1.5 cm and 1.5 × 1.2 cm TEE: transesophageal echocardiography

An African American man, aged 56, initially presented to the hospital due to fluid coming out of the pacemaker site, fever, chills, and diarrhea. He had a previously diagnosed history of hypertension, diabetes mellitus, chronic kidney disease, heart failure with reduced ejection fraction, and recurrent implantable cardio-defibrillator infections. On initial workup, the blood culture grew *P. aeruginosa*. Subsequently, the lead extraction procedure was performed that revealed vegetations on the atrial and the ventricular aspect of the tricuspid valve with the help of transesophageal echocardiography (TEE) (Figure 1).

**Figure 2:**
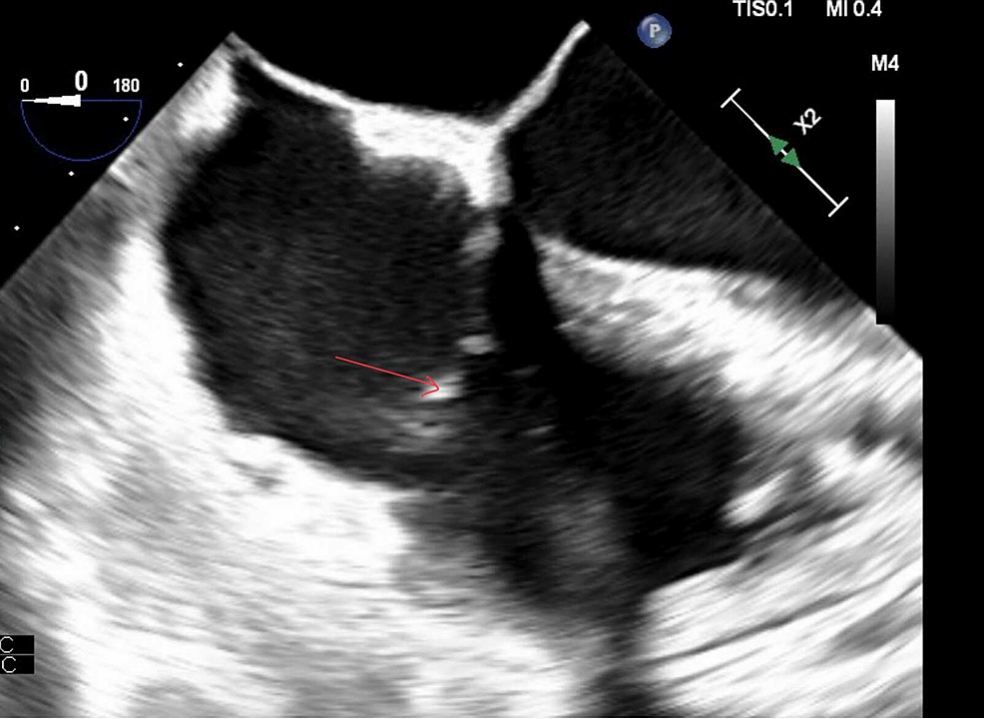
TEE showing significant reduction (>80%) of vegetation TEE: transesophageal echocardiography

A decision was taken to approach with a minimally invasive approach for removal of the vegetations with AngioVac assistance, due to comorbidities making him a high-risk surgical case. The purpose of this procedure was to reduce the vegetation size and decrease the risk of emboli with effective antibiotic penetration. To begin with, access was obtained at the right common femoral vein and the right internal jugular vein (IJV). The venotomy was serially dilated followed by placement of a 19 French size (Fr) reinfusion cannula through the IJV. The venotomy was serially dilated followed by placement of a Gore (Gore & Associates, Inc., Newark) 26 Fr dry seal sheath into the inferior vena cava. The AngioVac circuit was started, and debulking of the tricuspid valve vegetation was done under TEE with fluoroscopic guidance. TEE showed a significant reduction in the vegetation of the tricuspid valve (Figure 2).

After that, the AngioVac system was stopped, and the filtered blood from the circuit was returned to the patient by perfusion. Postoperatively, no complications were observed, and the patient remained hemodynamically stable. The repeat transthoracic echocardiography (TTE) showed mild tricuspid regurgitation with no other complications. He was discharged home with 6 weeks of intravenous cefepime outpatient treatment as per infectious disease recommendation. He was given a follow-up appointment but did not show up.

Case two

**Figure 3:**
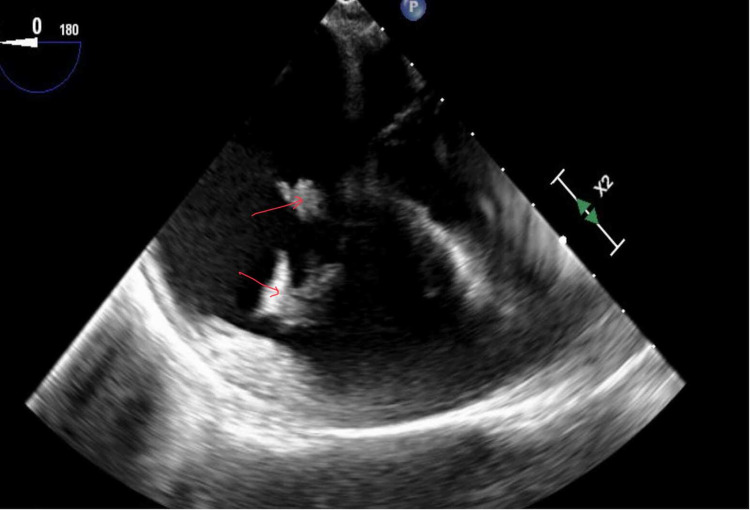
TEE showing lesions on the anterior and septal leaflet of the tricuspid valve TEE: transesophageal echocardiography

A 39-year-old woman with a history of hepatitis C and intravenous drug use presented to the hospital with septic shock secondary to IE. She was found to have two large vegetations with methicillin-resistant *S. aureus* bacteremia and severe tricuspid regurgitation. The lesion on the anterior leaflet measured 1.5 × 0.9 cm, and the lesion on the septal tricuspid valve leaflet measured 0.8 × 0.4 cm with the help of TEE (Figure 3).

**Figure 4:**
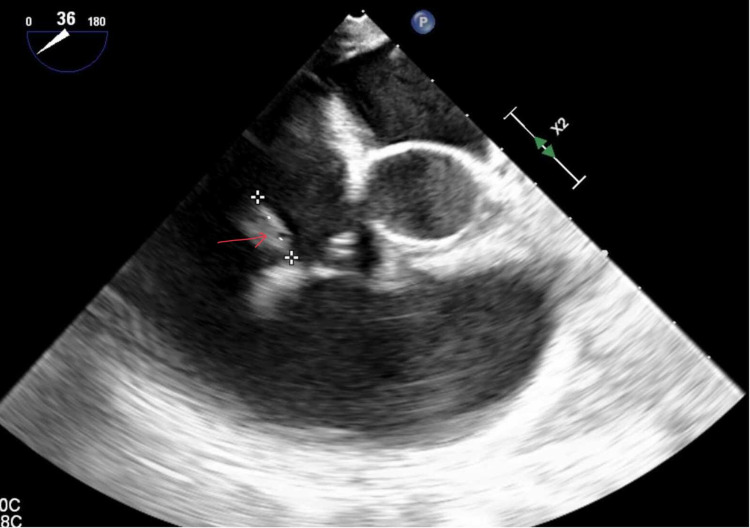
TEE demonstrates reduction of vegetations on the anterior and the septal leaflet TEE: transesophageal echocardiography

A decision was taken to approach with a minimally invasive approach for removal of the vegetations with AngioVac assistance due to critical ill status. A similar procedural approach was taken to advance the AngioVac catheter into the right atrium and debulking of the vegetations was performed. TEE demonstrated a significant reduction, greater than 80% debulking of the septal leaflet vegetations and 50% reduction of the anterior leaflet vegetation (Figure 4).

Postoperatively, no complications were observed, and the patient remained hemodynamically stable. The repeat TTE showed a significant decrease in vegetation size with no complications. She was discharged to inpatient rehabilitation with 4 weeks of intravenous antibiotic treatment as per infectious disease recommendation. The follow-up appointment was scheduled but she did not show up.

Case three

**Figure 5:**
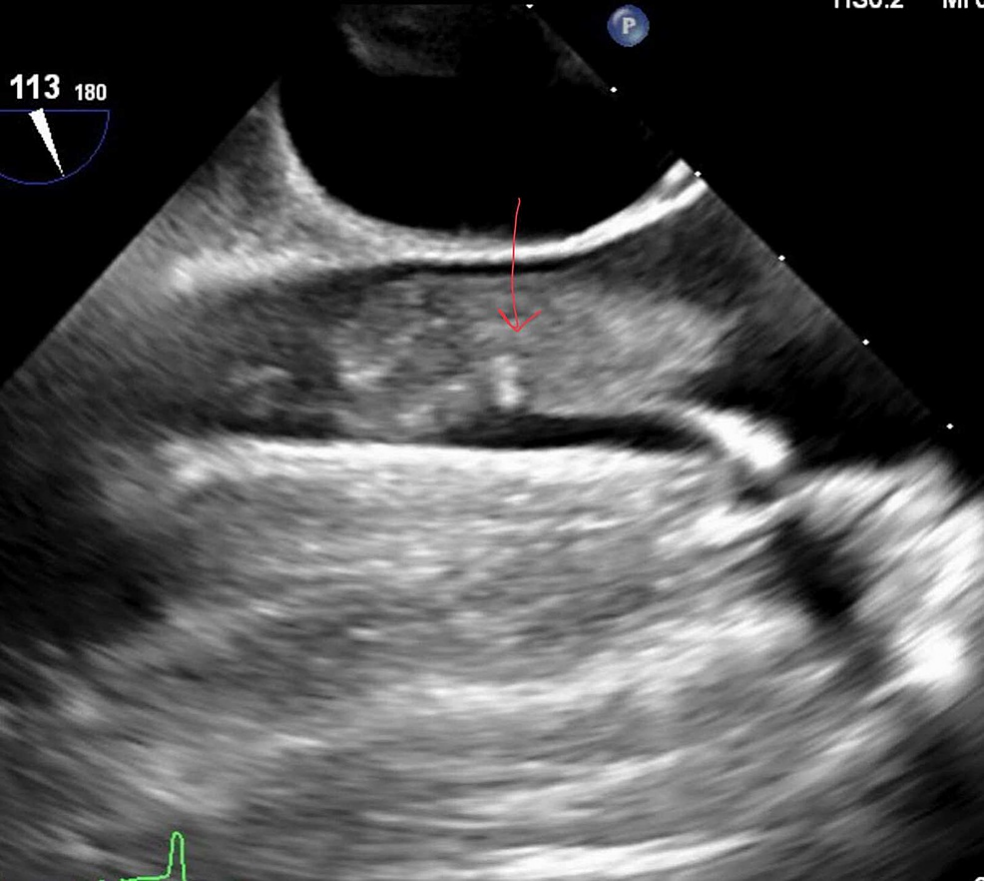
TEE indicates 2.7 × 1.0 cm vegetation in the superior vena cava TEE: transesophageal echocardiography

Woman, aged 70, with a history of coronary artery disease with stents, diabetes mellitus, hypertension, end-stage renal disease on hemodialysis, sick sinus syndrome status post pacemaker placement, and peripheral arterial disease presented to the hospital with sepsis and was found to have methicillin-sensitive *S. aureus* bacteremia. Further investigation revealed a large 2.7 × 1.0 cm echo-density in the superior vena cava (SVC), 0.9 × 0.1 cm mobile echo-density on the pacemaker leads, and vegetation at the tip of the tunneled catheter under TEE (Figure 5).

**Figure 6:**
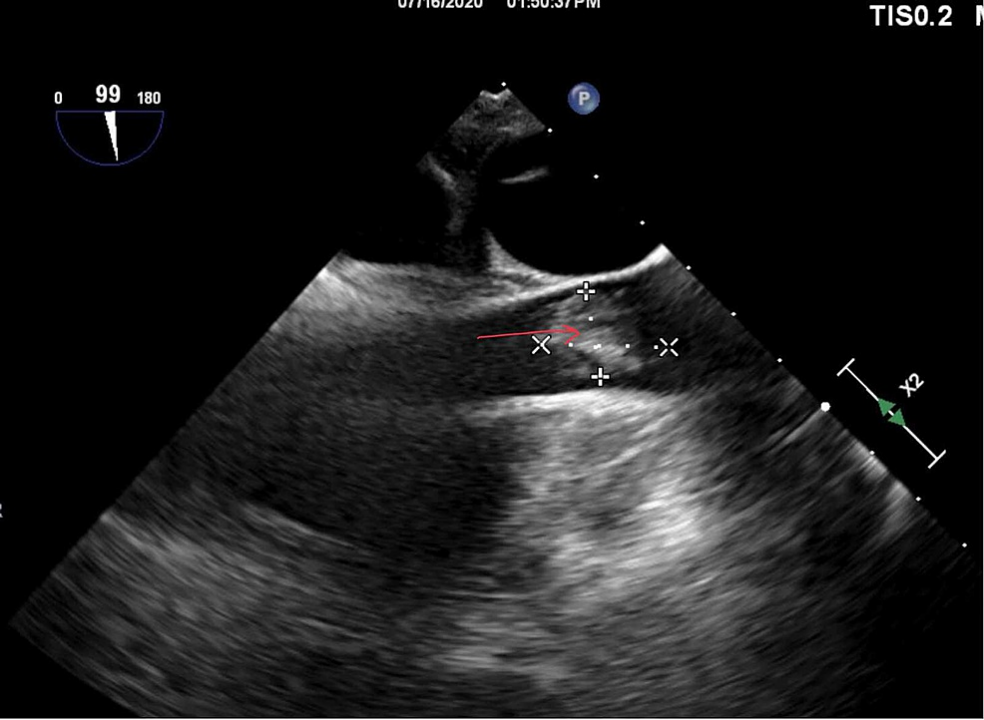
TEE showing greater than 70% debulking of the superior vena cava vegetation TEE: transesophageal echocardiography

A decision was taken to approach with a minimally invasive approach for removal of the vegetations with AngioVac assistance due to patient’s comorbidities. As mentioned in previous cases, the purpose of this procedure was to decrease the vegetation, and a similar procedural approach was taken to debulk the vegetation from all three sources. After that, TEE demonstrated a significant reduction, greater than 70% debulking of the SVC vegetation with the removal of pacemaker leads and replacement of tunneled catheter with the triple lumen central line (Figure 6).

Postoperatively, no complications were observed, and the patient remained hemodynamically stable. The repeat TTE showed mild to moderate tricuspid regurgitation with no pericardial effusion. She was discharged home with 6 weeks of intravenous nafcillin treatment as per infectious disease recommendation. During the follow-up appointment, she was feeling much better with no additional complaints.

## Discussion

In patients with right-sided IE, tricuspid valve endocarditis is more common than pulmonic valve endocarditis. Fever is the most common symptom, in addition to headache, malaise, chills, dyspnea, night sweats, abdominal pain, and weight loss. Moreover, septic pulmonary emboli are common in patients with tricuspid involvement (up to 75% cases) having clinical manifestations of cough, hemoptysis, dyspnea, and pleuritic chest pain [8]. The cardiac examination should include any heart murmurs or jugular venous distension, and the pulmonary examination should include any variations in breath sounds. A focal neuro examination should be performed, and a thorough abdominal examination should include pain related to an infarction or septic emboli. The most common imaging is chest radiography which usually demonstrates septic pulmonary emboli in more than half of cases, in addition to abscesses and pleural effusions [9].

The initial evaluation for patients of IE includes blood cultures and TEE to visualize the vegetation burden. Blood cultures from three different sites (spaced 30-60 minutes from each other) are obtained prior to the initiation of antibiotic therapy. Successful management of tricuspid endocarditis requires parenteral antibiotic therapy and removal of any indwelling intravascular devices, such as the infected pacemaker lead and the tunneled catheter. Some common indications for the surgical approach to remove vegetations include immense vegetation size (>20 mm), recurrent septic pulmonary emboli, highly resistant organisms, or persistent bacteremia despite targeted antibiotics [10]. On the contrary, patients who are considered at high risk for surgery due to comorbidities or vegetation burden opt for an alternative approach.

The AngioVac device uses a venovenous bypass circuit for percutaneous thrombectomy [11]. It has been applied in the setting of iliocaval, pulmonary embolus, and right heart thrombus in patients who fail medical therapy or have high surgical risk [11]. The study found that iliocaval and right heart thrombi were most amenable to AngioVac thrombectomy with 100% and 60% success rates, respectively [11]; while pulmonary embolus had a 33% success rate [11]. Another study reports the AngioVac device case series describing the outcomes in evacuating large caval thrombi or intracardiac masses in pulmonary embolism with no pulmonary hemorrhages, strokes, or myocardial infarctions [2]. Finally, a case report was published using the AngioVac aspiration device to remove a right atrial thrombus from a high surgical risk patient for open removal with no complications confirming the safety and efficacy of this procedure for the treatment of unwanted intracardiac and intravascular masses [12]. Hence, our case illustrates the integral use of the AngioVac device in the medical field.

## Conclusions

The AngioVac device is used to remove a variety of materials, such as vegetations, thrombi, or emboli. IE is a prevalent disease, with tricuspid valve involvement being more common in intravenous drug users and patients with infected intravascular lines. Treatment varies depending on the surgical risk and severity of the disease. Utilizing the AngioVac system has opened an alternative approach for high surgical risk patients. Thus, our case series illustrates debulking vegetations in tricuspid endocarditis and vena cava using the AngioVac aspiration system.
